# Supplementary material for: Evaluating Population Normalization Methods Using Chemical Data for Wastewater-Based Epidemiology: Insights from a Site-Specific Case Study
Source: Viruses. 2025 May 4;17(5):672. doi: 10.3390/v17050672 (PMC12115702; doi:10.3390/v17050672)
Supplement: Supplementary file 1 [file viruses-17-00672-s001.zip › viruses-3557852-supplementary.pdf]

Supplementary material

Evaluating Population Normalization Methods Using Chemical Data for Wastewater-Based Epidemiology: Insights from a Site-Specific Case Study

Marco Verani [1,†], Ileana Federigi [1,*,†], Alessandra Angori [1], Alessandra Pagani [1], Francesca Marvulli [2], Claudia Valentini [3], Nebiyu Tariku Atomsa [1], Beatrice Conte [1] and Annalaura Carducci [1]

[1] Laboratory of Hygiene and Environmental Virology, Department of Biology, University of Pisa, Via S. Zeno 35/39, 56123 Pisa, Italy; marco.verani@unipi.it (M.V.); ileana.federigi@unipi.it (I.F.); alessandra.angori@biologia.unipi.it (A.A.); alessandra.pagani@phd.unipi.it (A.P.); nebiyu.atomsa@phd.unipi.it (N.T.A.); beatrice.conte@biologia.unipi.it (B.C.); annalaura.carducci@unipi.it (A.C.)
[2] Acque S.p.A., Via A. Bellatalla 1, 56121 Pisa, Italy; f.marvulli@acque.net (F.M.)
[3] Gaia S.p.A., Via G. Donizetti 16, 55045 Pietrasanta, Italy; claudia.valentini@gaia-spa.it (C.V.)
* Correspondence: ileana.federigi@unipi.it; Tel.: +39-050-2213645
† These authors contributed equally to this work.

List of tables

Table S1. Environmental data from February 2021 to March 2023 (n = 359 sampling date). For each sampling date, hydrochemical parameters (flow rate, chemical oxygen demand - COD, biochemical oxygen demand - BOD_5, and ammonia NH_4-N) and viral data (SARS-CoV-2) are reported, separately for each WWTP.

Table S2. Clinical surveillance data from February 2021 to Decembre 2021 in the catchment area of WWTP1 (n = 18) referred to the weeks when SARS-CoV-2 monitoring was performed. For each week, the total number of new COVID-19 cases is reported for the cities served by WWTP1 (three municipalities), along with the average weekly adjusted clinical cases (ACC), calculated by considering the fraction of each municipality's population served by the WWTP, as described in Eq. 5 of the main text.

Table S3. Clinical surveillance data from February 2021 to Decembre 2021 in the catchment area of WWTP2 (n = 19) referred to the weeks when SARS-CoV-2 monitoring was performed. For each week, the total number of new COVID-19 cases is reported for the cities served by WWTP2 (six municipalities), along with the average weekly adjusted clinical cases (ACC), calculated by considering the fraction of each municipality's population served by the WWTP, as described in Eq. 5 of the main text.

Table S4. Clinical surveillance data from February 2021 to Decembre 2021 in the catchment area of WWTP3 (n = 10) referred to the weeks when SARS-CoV-2 monitoring was performed. For each week, the total number of new COVID-19 cases is reported for the cities served by WWTP3 (four municipalities), along with the average weekly adjusted clinical cases (ACC), calculated by considering the fraction of each municipality's population served by the WWTP, as described in Eq. 5 of the main text.

Table S5. Clinical surveillance data from February 2021 to Decembre 2021 in the catchment area of WWTP4 (n = 17) referred to the weeks when SARS-CoV-2 monitoring was performed. For each week, the total number of new COVID-19 cases is reported for the cities served by WWTP4 (four municipalities), along with the average weekly adjusted clinical cases (ACC), calculated by considering the fraction of each municipality's population served by the WWTP, as described in Eq. 5 of the main text.

List of Figures

Figure S1. Spearman correlations among chemical parameters, flow rate and rainfall in WWTP1 (n = 91) (a), WWTP2 (n = 93) (b), WWTP3 (n = 85) (c), WWTP4 (n = 90) (d). Colors represent the value of Spearman's ρ correlation: the darker the color, the larger the correlation magnitude. Asterisk indicates statistical significance at 0.05 level.

Figure S2. Relationship between precipitation in the previous 24 h (independent variable) and wastewater flowrate across four wastewater treatment plants (WWTPs), analyzed using ordinary least squares (OLS, blue line) and generalized least squares (GLS, red dotted line) regressions, separately for each WWTP: (a) WWTP1, (b) WWTP2, (c) WWTP3, (d) WWTP4. Each graph displays the model fit, coefficient of determination (R^2), and Durbin-Watson (DW) statistic to assess autocorrelation of residuals. GLS was applied in cases where significant autocorrelation was detected (DW < 2)

Figure S3. Relationship between wastewater flowrate (independent variable) and effluent concentrations of COD, BOD_5, and NH_4^+-N across four wastewater treatment plants (WWTPs), analyzed using ordinary least squares (OLS, blue line) and generalized least squares (GLS, red dotted line) regressions: (a) WWTP1, (b) WWTP2, (c) WWTP3, (d) WWTP4, (e) Pooled data. Each graph displays the model fit, coefficient of determination (R^2), and Durbin-Watson (DW) statistic to assess autocorrelation of residuals. GLS was applied in cases where significant autocorrelation was detected (DW < 2).

Table S1. Environmental data from February 2021 to March 2023 (n = 359 sampling date). For each sampling date, hydrochemical parameters (flow rate, chemical oxygen demand - COD, biochemical oxygen demand - BOD_5, and ammonia NH_4-N) and viral data (SARS-CoV-2) are reported, separately for each WWTP.

Sampling date	Flow rate (L/day)	COD (mg/L)	BOD_5 (mg/L)	NH_4-N (mg/L)	SARS-CoV-2 (GC/L)
**WWTP1**					
02/02/2021	21298111	161	46	23	Not sampled
04/02/2021	16165376	74,3	26	16	Not sampled
09/02/2021	24313697	103	36	18	< 3
11/02/2021	18246604	77,9	15	16	Not sampled
16/02/2021	24413468	149	52	29	< 3
18/02/2021	15146032	103	50	22	Not sampled
23/02/2021	10564212	217	92	38	Not sampled
25/02/2021	9839393	159	87	36	Not sampled
02/03/2021	10066540	261	80	44	< 3
04/03/2021	8615815	233	77	42	Not sampled
09/03/2021	8775200	250	78	49	Not sampled
11/03/2021	9355685	217	67	46	Not sampled
15/03/2021	8022053	284	140	53	< 3
18/03/2021	8124151	446	200	42	Not sampled
22/03/2021	9011389	328	160	54	75068
25/03/2021	9521366	251	82	52	Not sampled
30/03/2021	10648626	317	130	59	< 3
01/04/2021	12786742	562	170	67	Not sampled
07/04/2021	8523617	346	150	48	Not sampled
08/04/2021	8499351	316	130	58	Not sampled
13/04/2021	13229474	160	93	24	97611
15/04/2021	10778462	220	110	38	Not sampled
21/04/2021	9440900	288	130	56	97273
05/10/2021	10080000	261	120	49	2925
12/10/2021	9912000	149	88	43	< 3
19/10/2021	9336000	291	82	60	< 3
26/10/2021	9336000	432	140	49	7750
02/11/2021	11208000	319	96	53	2725
09/11/2021	9624000	258	99	53	4900
15/11/2021	18936000	109	49	11	< 3
22/11/2021	12672000	239	97	49	< 3
29/11/2021	11616000	196	78	42	4950
06/12/2021	17544000	63,9	20	17	< 3
19/01/2022	13584000	503	280	35	69250
25/01/2022	11976000	263	130	46	135250
01/02/2022	10968000	445	190	44	20650

08/02/2022	11544000	427	150	50	54000
15/02/2022	13416000	245	95	47	< 3
22/02/2022	13848000	177	90	40	< 3
01/03/2022	12216000	234	100	41	10350
07/03/2022	9768000	325	130	49	20950
14/03/2022	12240000	314	150	57	2625
22/03/2022	10104000	281	120	50	42925
30/03/2022	10344000	309	140	59	46750
05/04/2022	14544000	229	88	34	44900
13/04/2022	12048000	262	110	52	< 3
19/04/2022	10128000	254	120	54	33233
27/04/2022	14256000	345	170	50	29995
03/05/2022	12672000	355	160	44	56201
10/05/2022	12312000	243	100	47	77423
17/05/2022	11280000	295	94	56	35206
23/05/2022	10176000	339	130	60	7516
31/05/2022	10320000	295	100	57	< 3
08/06/2022	10104000	572	240	64	29200
14/06/2022	9048000	276	81	40	7373
21/06/2022	8568000	496	170	56	< 3
28/06/2022	9048000	597	190	54	9168
05/07/2022	10008000	276	90	52	92044
12/07/2022	9192000	439	140	58	< 3
18/07/2022	8160000	759	250	60	< 3
26/07/2022	8472000	1220	440	61	< 3
02/08/2022	8339000	1260	410	59	< 3
08/08/2022	6694000	334	100	50	Not sampled
16/08/2022	7224000	594	210	56	< 3
24/08/2022	8640000	307	110	51	< 3
29/08/2022	8856000	261	87	50	14919
06/09/2022	10872000	397	190	51	21222
13/09/2022	7872000	1640	700	56	8544
20/09/2022	9432000	312	180	61	65964
27/09/2022	10236000	242	110	52	35206
04/10/2022	9360000	311	130	64	89715
11/10/2022	9204000	299	140	66	46971
18/10/2022	8472000	371	150	68	99400
25/10/2022	7992000	429	190	73	64708
02/11/2022	7920000	369	180	64	236064
09/11/2022	9864000	269	120	59	109077
14/11/2022	10488000	255	110	53	< 3
21/11/2022	10464000	240	150	46	83076
28/11/2022	11688000	167	78	37	33554

06/12/2022	15504000	266	120	35	80974
14/12/2022	13560000	537	260	40	82810
20/12/2022	15504000	236	120	36	145996
10/01/2023	15888000	196	91	19	64295
18/01/2023	14016000	77,6	34	20	56381
24/01/2023	13344000	191	68	33	147405
01/02/2023	10560000	434	270	50	22410
06/02/2023	8880000	384	160	54	456699
13/02/2023	8520000	283	120	58	14449
22/02/2023	9024000	713	330	87	< 3
27/02/2023	11376000	131	40	22	323127
08/03/2023	11208000	237	120	33	156155
**WWTP2**					
01/02/2021	27240306	56,4	25	7	Not sampled
04/02/2021	33080237	115	26	22	< 3
09/02/2021	33035073	108	51	16	Not sampled
10/02/2021	38830341	47,9	21	11	Not sampled
11/02/2021	30362700	112	49	24	Not sampled
16/02/2021	26683577	189	65	37	< 3
17/02/2021	28485038	164	46	36	Not sampled
22/02/2021	22392763	129	50	30	Not sampled
25/02/2021	19888538	158	49	41	Not sampled
01/03/2021	18470700	122	62	36	Not sampled
03/03/2021	18007950	137	47	42	< 3
08/03/2021	17962200	128	50	40	Not sampled
10/03/2021	18375413	288	92	46	Not sampled
16/03/2021	16893263	237	120	55	< 3
18/03/2021	16634738	226	110	47	Not sampled
23/03/2021	13806750	205	75	52	97375
24/03/2021	15616838	278	130	48	Not sampled
29/03/2021	16064025	182	55	47	97409
31/03/2021	17474588	232	92	48	Not sampled
06/04/2021	18029438	141	56	47	126354
07/04/2021	14103038	400	200	48	Not sampled
13/04/2021	30733681	108	78	15	< 3
15/04/2021	17046938	0	52	32	Not sampled
20/04/2021	15691088	284	72	52	< 3
22/04/2021	15675150	181	42	44	Not sampled
04/10/2021	13344000	386	56	43	< 3
13/10/2021	13488000	236	85	50	< 3
19/10/2021	13248000	184	46	53	< 3
25/10/2021	12576000	158	45	53	4650
02/11/2021	14784000	287	87	34	< 3

08/11/2021	16512000	132	98	30	< 3
16/11/2021	18912000	158	63	24	5325
22/11/2021	13128000	150	64	46	5025
30/11/2021	15288000	142	52	40	< 3
07/12/2021	18744000	126	40	37	9000
18/01/2022	15312000	110	45	42	37750
24/01/2022	14424000	143	62	47	5200
01/02/2022	13728000	192	77	55	9275
08/02/2022	13464000	305	100	47	12925
14/02/2022	13296000	195	66	50	< 3
21/02/2022	13440000	181	64	44	< 3
28/02/2022	13482000	171	88	43	< 3
08/03/2022	12576000	243	81	59	10100
15/03/2022	12288000	344	120	64	< 3
21/03/2022	12048000	306	130	61	< 3
29/03/2022	11544000	330	99	65	< 3
05/04/2022	16944000	257	77	34	< 3
12/04/2022	12672000	254	87	55	< 3
19/04/2022	10968000	269	170	51	< 3
27/04/2022	19152000	Not determined	Not determined	27	22054
03/05/2022	12864000	289	130	45	4739
11/05/2022	13464000	287	99	43	12469
18/05/2022	12600000	281	99	46	13041
23/05/2022	11448000	200	61	52	9837
31/05/2022	11280000	180	67	52	< 3
07/06/2022	11472000	303	100	58	< 3
15/06/2022	12048000	329	140	55	8822
21/06/2022	11088000	293	93	58	< 3
29/06/2022	11712000	229	57	47	28496
05/07/2022	10800000	240	81	51	< 3
13/07/2022	11856000	404	120	50	< 3
20/07/2022	9237000	254	75	55	16319
26/07/2022	10680000	285	90	50	< 3
02/08/2022	11427000	252	85	58	5117
09/08/2022	11180000	170	45	46	< 3
17/08/2022	18979000	72,1	24	13	< 3
24/08/2022	10920000	210	79	82	< 3
31/08/2022	11520000	108	31	43	10093
06/09/2022	11472000	172	51	51	< 3
14/09/2022	11856000	243	110	40	< 3
21/09/2022	12048000	280	84	46	3960
27/09/2022	13032000	162	45	34	12711
05/10/2022	12336000	208	92	44	21913

12/10/2022	12216000	239	84	41	21773
19/10/2022	11736000	221	75	50	37777
25/10/2022	11580000	216	130	56	< 3
02/11/2022	11376000	161	72	51	26052
08/11/2022	11388000	147	62	52	13423
16/11/2022	15180000	177	83	39	< 3
23/11/2022	22320000	49	21	10	13294
30/11/2022	12096000	257	130	53	6741
06/12/2022	20568000	55,7	27	9,3	10388
13/12/2022	22941600	219	110	30	24592
20/12/2022	21360000	108	52	33	22196
11/01/2023	22296000	107	48	27	204372
18/01/2023	21528000	65,7	32	45	29049
24/01/2023	20520000	76,2	22	21	7421
31/01/2023	15960000	389	170	41	5761
08/02/2023	14520000	250	110	56	8654
13/02/2023	14136000	259	100	50	10256
22/02/2023	14232000	394	200	49	13294
01/03/2023	16416000	165	66	43	216504
07/03/2023	18888000	262	120	43	Not sampled
**WWTP3**					
16/02/2021	30790000	373	180	18	44599
25/02/2021	24917000	450	160	22	57862
09/03/2021	21607000	379	270	26	97392
03/06/2021	21528000	257	160	31	< 3
16/06/2021	20410000	384	290	30	< 3
21/09/2021	25647000	333	180	58	Not sampled
28/09/2021	20927000	274	190	33	Not sampled
07/10/2021	25184000	248	145	51	< 3
11/10/2021	18416000	275	150	52	Not sampled
19/10/2021	17025000	341	220	64	Not sampled
26/10/2021	17679000	460	370	53	Not sampled
02/11/2021	20651000	245	170	38	Not sampled
08/11/2021	20100000	450	310	54	Not sampled
16/11/2021	27969000	182	140	30	Not sampled
25/11/2021	20621000	294	250	41	6600
02/12/2021	24510000	293	160	15	7525
07/12/2021	22766000	98	62	23	< 3
13/12/2021	22500000	319	230	51	Not sampled
22/12/2021	19984000	406	230	51	18100
03/01/2022	24192000	297	200	43	Not sampled
13/01/2022	20673000	90,7	62	20	< 3
17/01/2022	20238000	422	200	57	Not sampled

25/01/2022	18724000	79,1	50	17	Not sampled
02/02/2022	18137000	207	180	37	< 3
09/02/2022	17002000	104	50	21	23400
14/02/2022	16160000	109	55	22	Not sampled
24/02/2022	17058000	403	140	70	< 3
02/03/2022	18323000	274	220	37	16000
07/03/2022	16587000	326	210	65	Not sampled
17/03/2022	15460000	464	300	51	22350
21/03/2022	15745000	395	290	60	Not sampled
29/03/2022	16843000	466	290	54	< 3
07/04/2022	25311000	424	220	42	Not sampled
12/04/2022	18321000	304	230	42	< 3
19/04/2022	17830000	416	360	43	< 3
27/04/2022	23659000	267	190	34	< 3
02/05/2022	19095000	310	250	36	Not sampled
10/05/2022	18387000	332	290	39	26388
18/05/2022	15906000	380	270	41	32185
26/05/2022	16284000	495	420	47	Not sampled
30/05/2022	16745000	311	210	46	Not sampled
06/06/2022	16160000	286	220	27	Not sampled
16/06/2022	16095000	411	280	45	Not sampled
20/06/2022	16371000	482	330	57	Not sampled
30/06/2022	18776000	345	250	39	Not sampled
04/07/2022	16356000	461	380	55	Not sampled
07/07/2022	16035000	439	380	52	Not sampled
12/07/2022	14760000	710	400	82	< 3
20/07/2022	16794000	436	340	55	83610
27/07/2022	15816000	503	460	53	< 3
03/08/2022	15387000	647	310	56	< 3
11/08/2022	16340000	480	350	54,5	Not sampled
17/08/2022	16726000	431	330	66	< 3
25/08/2022	16911000	328	300	56	Not sampled
31/08/2022	16073000	495	340	57	< 3
06/09/2022	16527000	287	220	71	< 3
13/09/2022	13875000	460	400	53	10158
19/09/2022	14206000	592	380	57	Not sampled
27/09/2022	13908000	486	400	54	70780
04/10/2022	13869000	405	320	54	151717
12/10/2022	13820000	432	390	63	197296
17/10/2022	13216000	442	390	64	Not sampled
26/10/2022	12330000	444	360	57	< 3
02/11/2022	11499000	630	340	64	170809
08/11/2022	11893000	555	400	70	264920

14/11/2022	13307000	453	350	50	Not sampled
22/11/2022	18903000	325	260	51	352320
29/11/2022	15627000	283	210	50	253301
06/12/2022	19663000	313	170	39	< 3
12/12/2022	23166000	294	145	37	Not sampled
19/12/2022	24773000	275	250	32	Not sampled
27/12/2022	20115000	313	280	54	25720
02/01/2023	24713000	213	180	24	Not sampled
10/01/2023	30654000	179	155	20	125187
17/01/2023	30812000	199	115	22	58779
24/01/2023	23676000	216	120	37	68330
30/01/2023	20363000	254	160	33	Not sampled
06/02/2023	20450000	249	140	32	Not sampled
14/02/2023	18451000	352	200	40	19589
23/02/2023	17333000	327	280	48	Not sampled
27/02/2023	26713000	226	170	42	Not sampled
07/03/2023	25027000	236	130	26	276186
14/03/2023	21563000	237	75	33	201125
22/03/2023	19574000	199	150	35	51215
28/03/2023	20451000	341	180	37	25474
**WWTP4**					
17/02/2021	18374000	289	220	31	< 3
24/02/2021	11481000	403	80	40	< 3
10/03/2021	9296000	490	400	53	< 3
24/03/2021	8103000	231	180	57	126354
07/04/2021	9094000	497	360	67	163928
05/05/2021	7525000	456	240	45	< 3
09/06/2021	9777000	625	460	42	< 3
16/06/2021	8048000	523	360	52	< 3
23/06/2021	12330000	470	320	43	< 3
04/08/2021	12489000	654	380	51	351
18/08/2021	12789000	522	450	55	27
01/09/2021	10776000	593	370	56	20
22/09/2021	10759000	469	300	42	Not sampled
27/09/2021	15052000	347	230	33	Not sampled
06/10/2021	14941000	312	180	28	6075
11/10/2021	8862000	483	360	43	Not sampled
21/10/2021	9177000	594	320	44	550
25/10/2021	9246000	600	360	46	Not sampled
03/11/2021	13732000	489	270	34	8825
08/11/2021	11504000	412	240	44	Not sampled
17/11/2021	16071000	299	190	28	< 3
29/11/2021	18276000	412	250	23	Not sampled

01/12/2021	12779000	209	145	42	< 3
06/12/2021	15463000	316	220	37	Not sampled
13/12/2021	13858000	265	120	34	Not sampled
20/12/2021	11477000	578	300	45	Not sampled
05/01/2022	20125000	155	120	29	18175
12/01/2022	12888000	459	320	37	61075
19/01/2022	11301000	438	300	20	59500
26/01/2022	10873000	385	320	46	63750
02/02/2022	10395000	606	360	49	41500
07/02/2022	10489000	518	270	49	Not sampled
17/02/2022	11321000	511	120	44	< 3
23/02/2022	10726000	689	280	47	4425
02/03/2022	10567000	573	370	52	< 3
10/03/2022	9138000	700	260	49	27000
15/03/2022	10513000	636	240	57	Not sampled
23/03/2022	9247000	521	330	45	< 3
28/03/2022	9039000	477	320	60	Not sampled
06/04/2022	13379000	365	250	43	< 3
11/04/2022	10389000	489	340	50	Not sampled
20/04/2022	11147000	511	270	49	< 3
28/04/2022	12398000	475	270	43	Not sampled
04/05/2022	10565000	487	370	41	< 3
11/05/2022	10719000	703	380	50	46077
18/05/2022	10480000	701	400	47	5052
23/05/2022	10609000	578	340	55	Not sampled
30/05/2022	11747000	406	240	46	Not sampled
09/06/2022	11624000	518	350	46	Not sampled
15/06/2022	10895000	463	350	47	14449
23/06/2022	12383000	511	410	46	Not sampled
29/06/2022	17161000	776	340	39	24750
05/07/2022	11473000	516	390	55	62268
11/07/2022	11404000	616	280	61	Not sampled
18/07/2022	11734000	570	380	23	Not sampled
25/07/2022	11690000	549	440	56	Not sampled
03/08/2022	10696000	483	360	50	< 3
08/08/2022	11671000	660	540	50	Not sampled
18/08/2022	12765000	445	290	43	< 3
24/08/2022	11783000	609	320	55	28496
29/08/2022	10801000	454	320	56	Not sampled
05/09/2022	10576000	678	400	58	Not sampled
14/09/2022	9740000	380	260	52	13552
20/09/2022	10028000	437	260	48	24909
26/09/2022	10087000	461	250	41	Not sampled

06/10/2022	7849000	1210	560	61	Not sampled
13/10/2022	7392000	622	420	63	Not sampled
19/10/2022	7039000	580	360	65	115923
25/10/2022	8046000	593	300	61	< 3
03/11/2022	8313000	565	400	60	206346
10/11/2022	10042000	486	450	51	Not sampled
16/11/2022	11612000	412	370	39	198564
21/11/2022	8867000	618	260	48	Not sampled
28/11/2022	8605000	377	250	48	Not sampled
14/12/2022	11217000	304	210	37	5817
19/12/2022	15358000	321	300	35	Not sampled
28/12/2022	13045000	347	280	37	< 3
04/01/2023	12601000	425	200	43	57108
09/01/2023	20330000	380	140	38	Not sampled
18/01/2023	18834000	336	140	27	14174
25/01/2023	12702000	392	200	35	25802
31/01/2023	11655000	451	380	37	27510
07/02/2023	10964000	529	400	39	97508
15/02/2023	11010000	352	320	45	20685
20/02/2023	11073000	549	430	50	Not sampled
28/02/2023	12022000	617	220	39	101654
08/03/2023	19130000	210	70	26	292580
15/03/2023	16336000	283	150	27	163842
23/03/2023	11756000	458	420	41	Not sampled
29/03/2023	10663000	498	390	45	35889

Table S2. Clinical surveillance data from February 2021 to Decembre 2021 in the catchment area of WWTP1 (n = 18) referred to the weeks when SARS-CoV-2 monitoring was performed. For each week, the total number of new COVID-19 cases is reported for the cities served by WWTP1 (three municipalities), along with the average weekly adjusted clinical cases (ACC), calculated by considering the fraction of each municipality's population served by the WWTP, as described in Eq. 5 of the main text.

Week	Pisa (total new cases per week)	San Giuliano Terme (total new cases per week)	Vecchiano (total new cases per week)	Average weekly ACC_{WWTP1}
06-14/02/2021	111	35	17	11.69
15-21/02/2021	121	27	6	12.14
01-07/03/2021	126	46	17	13.52
15-21/03/2021	104	54	9	11.80
22-28/03/2021	108	56	17	12.32
29-04/04/2021	114	56	20	12.89
12-18/04/2021	87	37	15	9.59
19-25/04/2021	63	50	23	8.06
04-10/10/2021	26	10	0	2.78
11-17/10/2021	23	3	4	2.24
18-24/10/2021	53	20	1	5.65
25-31/10/2021	46	15	6	4.86
01-7/11/2021	73	15	8	7.31
08-14/11/2021	49	22	4	5.41
15-21/11/2021	58	26	5	6.40
22-28/11/2021	56	21	7	6.03
29-5/12/2021	81	31	15	8.79
06-12/12/2021	101	27	31	10.58

Table S3. Clinical surveillance data from February 2021 to Decembre 2021 in the catchment area of WWTP2 (n = 19) referred to the weeks when SARS-CoV-2 monitoring was performed. For each week, the total number of new COVID-19 cases is reported for the cities served by WWTP2 (six municipalities), along with the average weekly adjusted clinical cases (ACC), calculated by considering the fraction of each municipality's population served by the WWTP, as described in Eq. 5 of the main text.

Week	Capraia (total new cases per week)	Cerreto Guidi (total new cases per week)	Empoli (total new cases per week)	Montelupo Fiorentino (total new cases per week)	Montespertoli (total new cases per week)	Vinci (total new cases per week)	Average weekly ACC_{WWTP2}
02-07/02/2021	25	14	75	23	19	29	7.86
15-21/02/2021	61	34	105	26	21	42	11.25
01-07/03/2021	21	21	115	36	36	47	11.93
15-21/03/2021	20	35	158	53	33	71	16.49
22-28/03/2021	19	38	163	50	30	53	16.46
29-04/04/2021	20	42	177	65	30	43	17.74
05-11/04/2021	19	25	109	48	30	36	11.38
12-18/04/2021	10	21	78	22	24	28	7.93
19-25/04/2021	13	11	58	19	12	29	6.17
04-10/10/2021	1	3	21	2	5	14	2.17
11-17/10/2021	2	7	17	13	8	7	1.91
18-24/10/2021	3	19	33	8	18	8	3.28
25-31/10/2021	11	12	43	11	9	10	4.29
01-07/11/2021	7	4	48	16	9	12	4.78
08-14/11/2021	4	6	35	8	6	11	3.47
15-21/11/2021	1	3	49	6	2	16	4.69
22-28/11/2021	12	10	51	16	4	16	5.20
29-05/12/2021	9	14	44	30	10	17	4.87
06-12/12/2021	7	15	57	37	6	11	5.96

Table S4. Clinical surveillance data from February 2021 to Decembre 2021 in the catchment area of WWTP3 (n = 10) referred to the weeks when SARS-CoV-2 monitoring was performed. For each week, the total number of new COVID-19 cases is reported for the cities served by WWTP3 (four municipalities), along with the average weekly adjusted clinical cases (ACC), calculated by considering the fraction of each municipality's population served by the WWTP, as described in Eq. 5 of the main text.

Week	Carrara (total new cases per week)	Forte dei Marmi (total new cases per week)	Massa (total new cases per week)	Montignoso (total new cases per week)	Average weekly ACC_{WWTP3}
15-21/02/2021	62	13	116	10	12.90
22-28/02/2021	102	13	158	28	18.53
08-14/03/2021	101	11	132	36	16.34
31-06/06/2021	10	0	5	3	0.91
14-20/06/2021	3	0	1	0	0.22
04-10/10/2021	13	2	11	3	1.57
22-28/11/2021	80	4	37	10	6.89
29-5/12/2021	142	3	88	9	14.05
06-12/12/2021	122	9	73	10	11.87
20-26/12/2021	371	72	390	39	50.71

Table S5. Clinical surveillance data from February 2021 to Decembre 2021 in the catchment area of WWTP4 (n = 17) referred to the weeks when SARS-CoV-2 monitoring was performed. For each week, the total number of new COVID-19 cases is reported for the cities served by WWTP4 (four municipalities), along with the average weekly adjusted clinical cases (ACC), calculated by considering the fraction of each municipality's population served by the WWTP, as described in Eq. 5 of the main text.

Week	Camaiore (total new cases per week)	Massarosa (total new cases per week)	Vecchiano (total new cases per week)	Viareggio (total new cases per week)	Average weekly ACC_{WWTP4}
15-21/02/2021	71	41	6	182	25.94
22-28/02/2021	79	59	17	268	38.19
08-14/03/2021	68	57	15	193	27.51
22-28/03/2021	122	67	17	215	30.65
05-11/04/2021	74	44	21	133	18.96
03-09/05/2021	47	20	12	32	4.57
07-13/06/2021	4	5	0	2	0.29
14-20/06/2021	4	1	0	2	0.29
21-27/06/2021	5	1	1	6	0.86
02-08/08/2021	34	34	26	96	13.69
16-22/08/2021	51	43	10	113	16.11
30-05/09/2021	24	8	10	36	5.13
04-10/10/2021	24	1	0	16	2.28
18-24/10/2021	12	4	1	23	3.28
01-7/11/2021	13	11	8	25	3.57
15-21/11/2021	8	14	5	40	5.70
29-5/12/2021	30	15	15	72	10.26

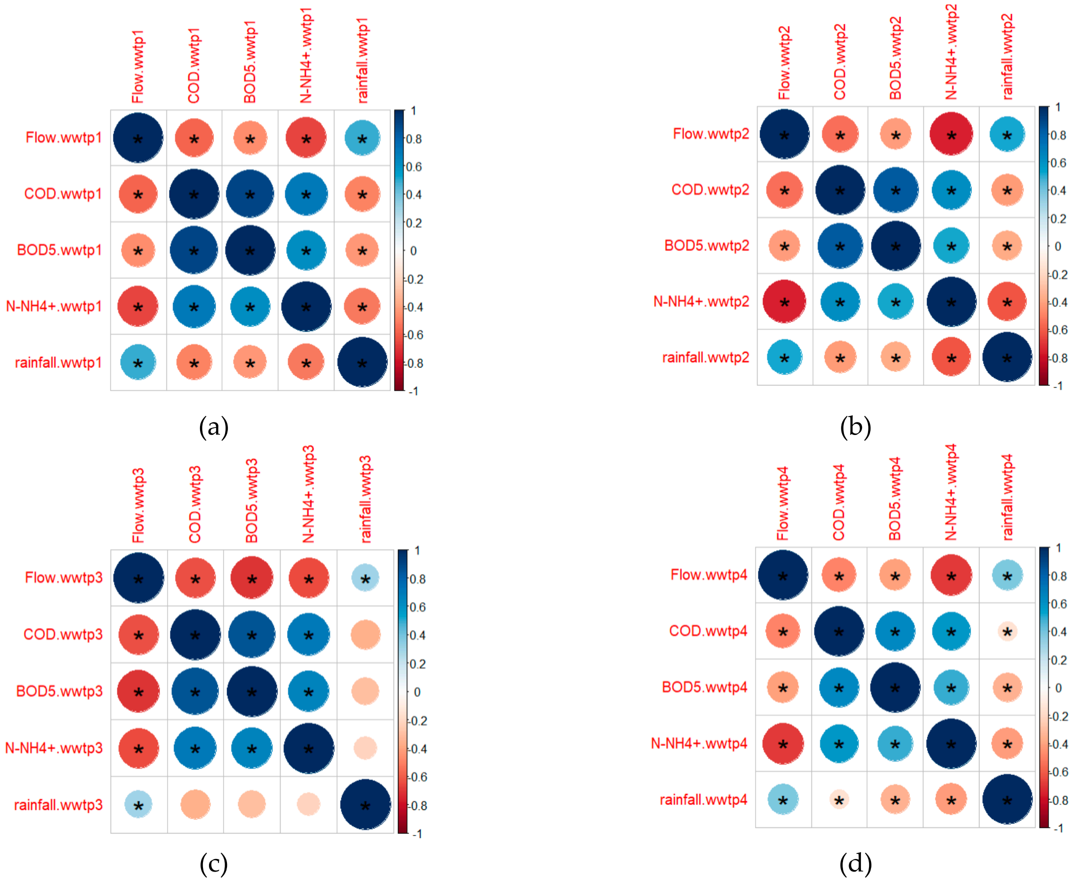

Figure S1. Spearman correlations among chemical parameters, flow rate and rainfall in WWTP1 (n = 91) (a), WWTP2 (n = 93) (b), WWTP3 (n = 85) (c), WWTP4 (n = 90) (d). Colors represent the value of Spearman's ϱ correlation: the darker the color, the larger the correlation magnitude. Asterisk indicates statistical significance at 0.05 level.

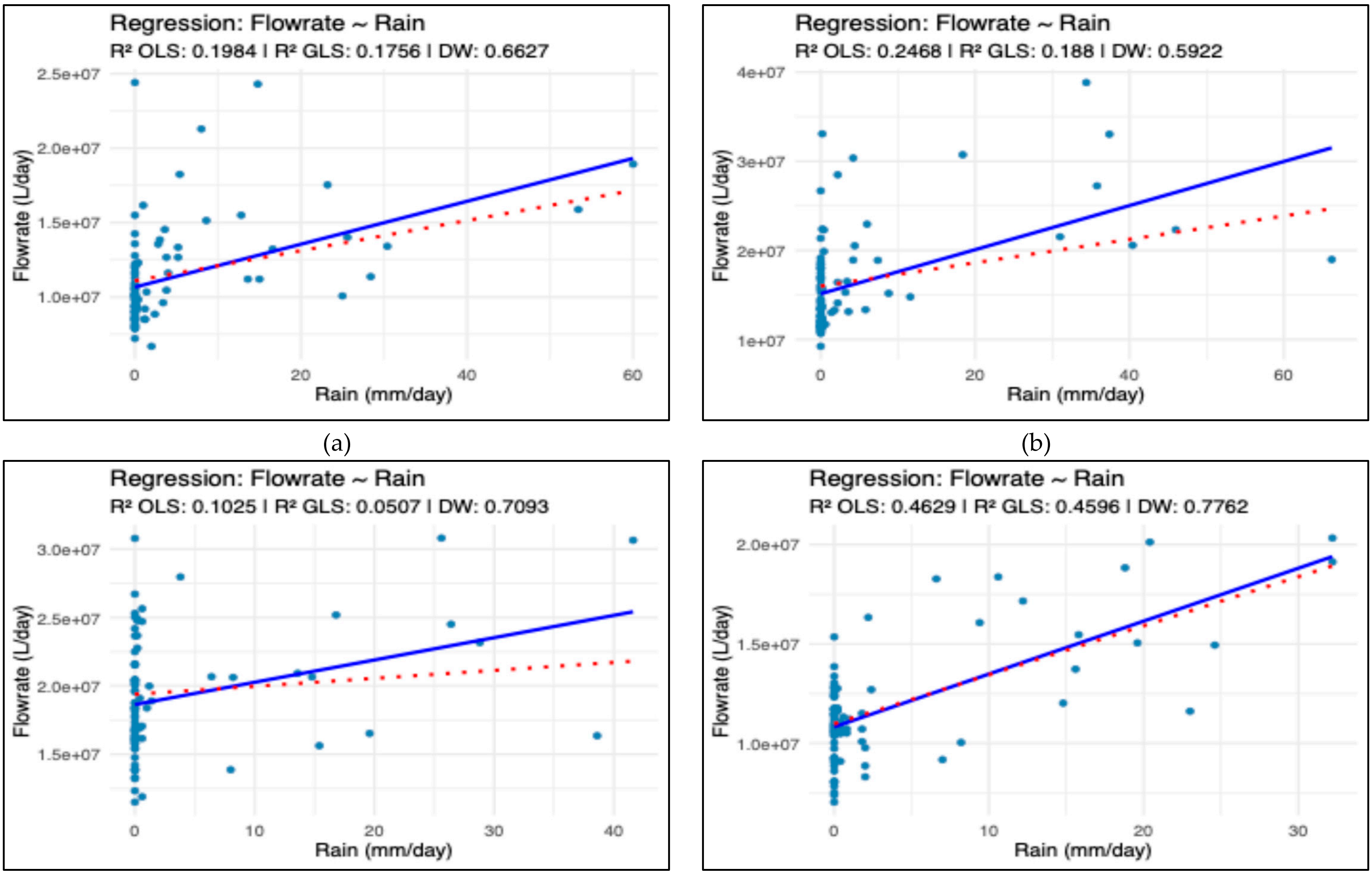

(a) (b) (c) (d)

Figure S2. Relationship between precipitation in the previous 24 h (independent variable) and wastewater flowrate across four wastewater treatment plants (WWTPs), analyzed using ordinary least squares (OLS, blue line) and generalized least squares (GLS, red dotted line) regressions, separately for each WWTP: (a) WWTP1, (b) WWTP2, (c) WWTP3, (d) WWTP4. Each graph displays the model fit, coefficient of determination (R^2), and Durbin-Watson (DW) statistic to assess autocorrelation of residuals. GLS was applied in cases where significant autocorrelation was detected (DW < 2).

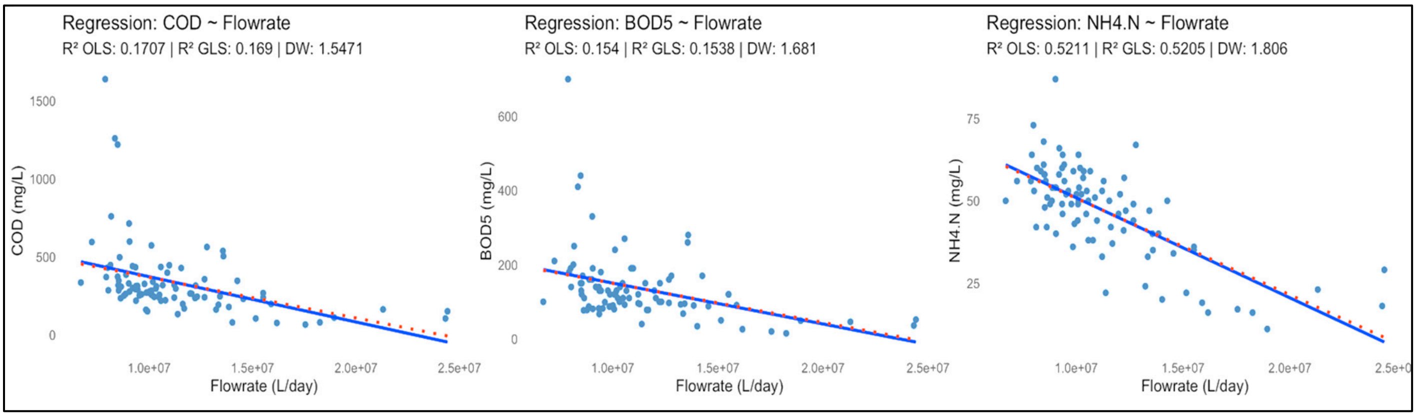
Regression: COD ~ Flowrate
R² OLS: 0.1707 | R² GLS: 0.169 | DW: 1.5471
COD (mg/L)
Flowrate (L/day)
Regression: BOD5 ~ Flowrate
R² OLS: 0.154 | R² GLS: 0.1538 | DW: 1.681
BOD5 (mg/L)
Flowrate (L/day)
Regression: NH4.N ~ Flowrate
R² OLS: 0.5211 | R² GLS: 0.5205 | DW: 1.806
NH4.N (mg/L)
Flowrate (L/day)

(a)

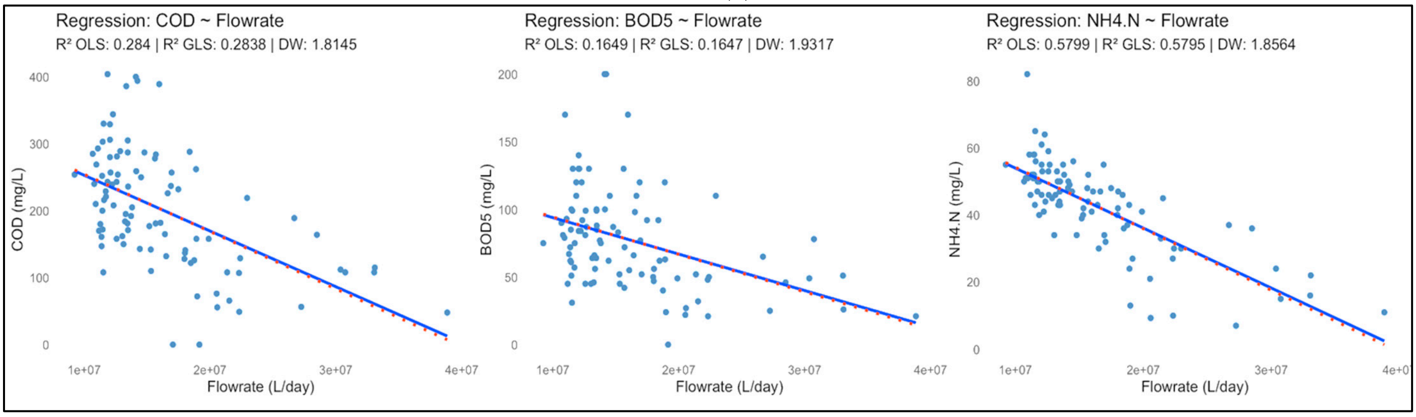
Regression: COD ~ Flowrate
R² OLS: 0.284 | R² GLS: 0.2838 | DW: 1.8145
COD (mg/L)
Flowrate (L/day)
Regression: BOD5 ~ Flowrate
R² OLS: 0.1649 | R² GLS: 0.1647 | DW: 1.9317
BOD5 (mg/L)
Flowrate (L/day)
Regression: NH4.N ~ Flowrate
R² OLS: 0.5799 | R² GLS: 0.5795 | DW: 1.8564
NH4.N (mg/L)
Flowrate (L/day)

(b)

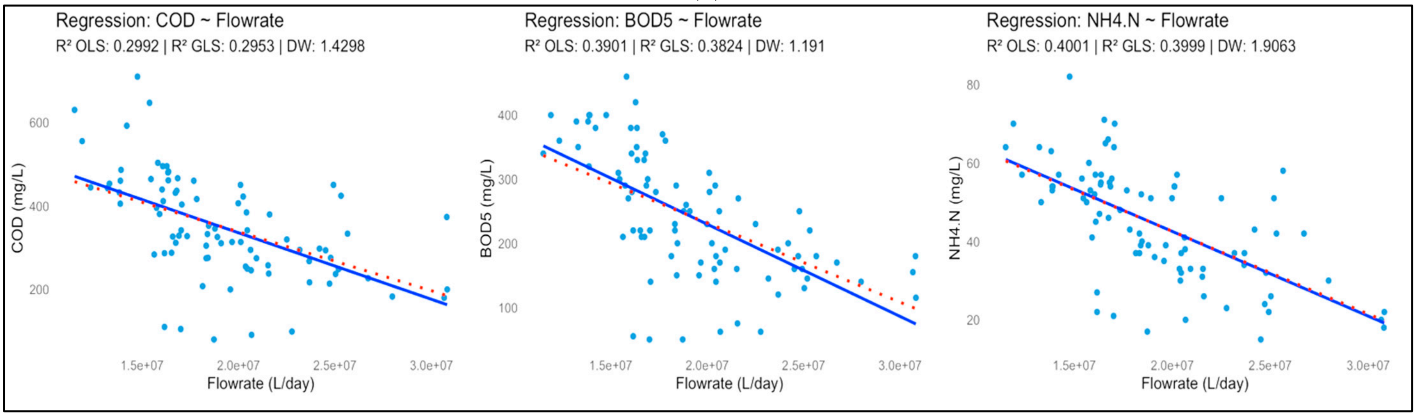
Regression: COD ~ Flowrate
R² OLS: 0.2992 | R² GLS: 0.2953 | DW: 1.4298
COD (mg/L)
Flowrate (L/day)
Regression: BOD5 ~ Flowrate
R² OLS: 0.3901 | R² GLS: 0.3824 | DW: 1.191
BOD5 (mg/L)
Flowrate (L/day)
Regression: NH4.N ~ Flowrate
R² OLS: 0.4001 | R² GLS: 0.3999 | DW: 1.9063
NH4.N (mg/L)
Flowrate (L/day)

(c)

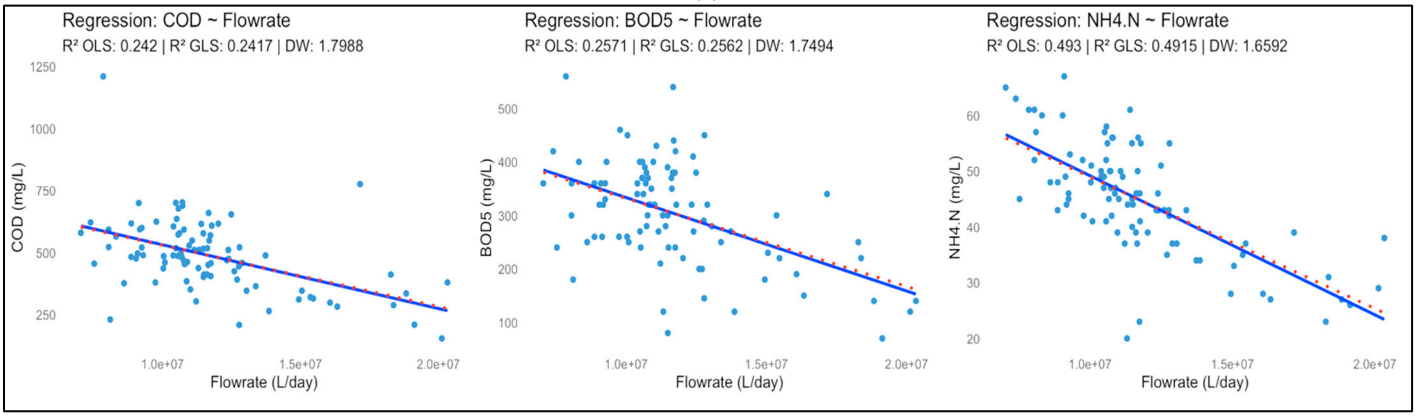
Regression: COD ~ Flowrate
R² OLS: 0.242 | R² GLS: 0.2417 | DW: 1.7988
COD (mg/L)
Flowrate (L/day)
Regression: BOD5 ~ Flowrate
R² OLS: 0.2571 | R² GLS: 0.2562 | DW: 1.7494
BOD5 (mg/L)
Flowrate (L/day)
Regression: NH4.N ~ Flowrate
R² OLS: 0.493 | R² GLS: 0.4915 | DW: 1.6592
NH4.N (mg/L)
Flowrate (L/day)

(d)

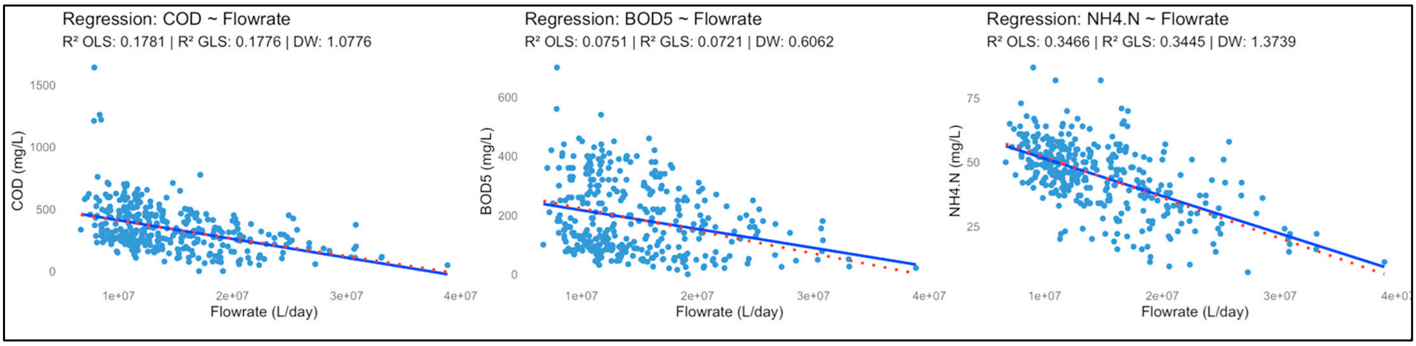

(e)

Figure S3. Relationship between wastewater flowrate (independent variable) and effluent concentrations of COD, BOD_5, and NH_4^+-N across four wastewater treatment plants (WWTPs), analyzed using ordinary least squares (OLS, blue line) and generalized least squares (GLS, red dotted line) regressions: (a) WWTP1, (b) WWTP2, (c) WWTP3, (d) WWTP4, (e) Pooled data. Each graph displays the model fit, coefficient of determination (R^2), and Durbin-Watson (DW) statistic to assess autocorrelation of residuals. GLS was applied in cases where significant autocorrelation was detected (DW < 2).
